# Molecular Evolution of the Oxygen-Binding Hemerythrin Domain

**DOI:** 10.1371/journal.pone.0157904

**Published:** 2016-06-23

**Authors:** Claudia Alvarez-Carreño, Arturo Becerra, Antonio Lazcano

**Affiliations:** 1 Facultad de Ciencias, Universidad Nacional Autónoma de México, Apdo. Postal 70–407, Cd. Universitaria, 04510, Mexico City, Mexico; 2 Miembro de El Colegio Nacional, Ciudad de México, México; California State University Fullerton, UNITED STATES

## Abstract

**Background:**

The evolution of oxygenic photosynthesis during Precambrian times entailed the diversification of strategies minimizing reactive oxygen species-associated damage. Four families of oxygen-carrier proteins (hemoglobin, hemerythrin and the two non-homologous families of arthropodan and molluscan hemocyanins) are known to have evolved independently the capacity to bind oxygen reversibly, providing cells with strategies to cope with the evolutionary pressure of oxygen accumulation. Oxygen-binding hemerythrin was first studied in marine invertebrates but further research has made it clear that it is present in the three domains of life, strongly suggesting that its origin predated the emergence of eukaryotes.

**Results:**

Oxygen-binding hemerythrins are a monophyletic sub-group of the hemerythrin/HHE (histidine, histidine, glutamic acid) cation-binding domain. Oxygen-binding hemerythrin homologs were unambiguously identified in 367/2236 bacterial, 21/150 archaeal and 4/135 eukaryotic genomes. Overall, oxygen-binding hemerythrin homologues were found in the same proportion as single-domain and as long protein sequences. The associated functions of protein domains in long hemerythrin sequences can be classified in three major groups: signal transduction, phosphorelay response regulation, and protein binding. This suggests that in many organisms the reversible oxygen-binding capacity was incorporated in signaling pathways. A maximum-likelihood tree of oxygen-binding hemerythrin homologues revealed a complex evolutionary history in which lateral gene transfer, duplications and gene losses appear to have played an important role.

**Conclusions:**

Hemerythrin is an ancient protein domain with a complex evolutionary history. The distinctive iron-binding coordination site of oxygen-binding hemerythrins evolved first in prokaryotes, very likely prior to the divergence of Firmicutes and Proteobacteria, and spread into many bacterial, archaeal and eukaryotic species. The later evolution of the oxygen-binding hemerythrin domain in both prokaryotes and eukaryotes led to a wide variety of functions, ranging from protection against oxidative damage in anaerobic and microaerophilic organisms, to oxygen supplying to particular enzymes and pathways in aerobic and facultative species.

## Introduction

Before the evolution of oxygenic photosynthesizers, sources of free oxygen were scarce [1]. Free molecular oxygen constitutes 21% of present-day terrestrial atmosphere and its main source is, essentially, oxygenic photosynthesis. Accumulation of free atmospheric oxygen during the Precambrian [1–3] is, undoubtedly, one of the major changes in the history of the planet and may be considered the most significant biogeochemical process after the origin of life itself. Oxygen-dependent metabolism evolved first in bacteria and is pervasive in contemporary eukaryotes.

The evolution of metazoans was constrained by the oxygen requirements of tissues [4–6]. Therefore, oxygen-carrier proteins that maintain a continuous delivery of oxygen while avoiding autoxidation as well as the formation and accumulation of reactive oxygen species [4] became essential for the development of animals. Four evolutionarily unrelated families of oxygen-carrier proteins are known: hemoglobin, hemerythrin and the two non-homologous families of molluscan and arthropodan hemocyanins. Such diverse assortment can only be understood in terms of the selective pressure imposed on the biosphere by free oxygen.

Hemerythrin is a small 118 amino acid protein classically studied in four metazoan phyla: Sipuncula, Brachiopoda, Priapulida and Annelida [7,8]; it has also been identified in other eukaryotes as well as in bacterial and archaeal genomes [9–12]. However, the molecular evolution of hemerythrin and its relationship to the geochemical history of the Earth has been largely ignored. In this work, we studied the phylogenetic distribution of hemerythrin-like sequences in 2521 completely sequenced bacterial, archaeal and eukaryotic genomes, and correlated the possible evolutionary scenarios with what is currently known about the structure and function of the hemerythrin domain in both prokaryotes and eukaryotes.

## Results

### Sequence similarity search

**Fig 1:**
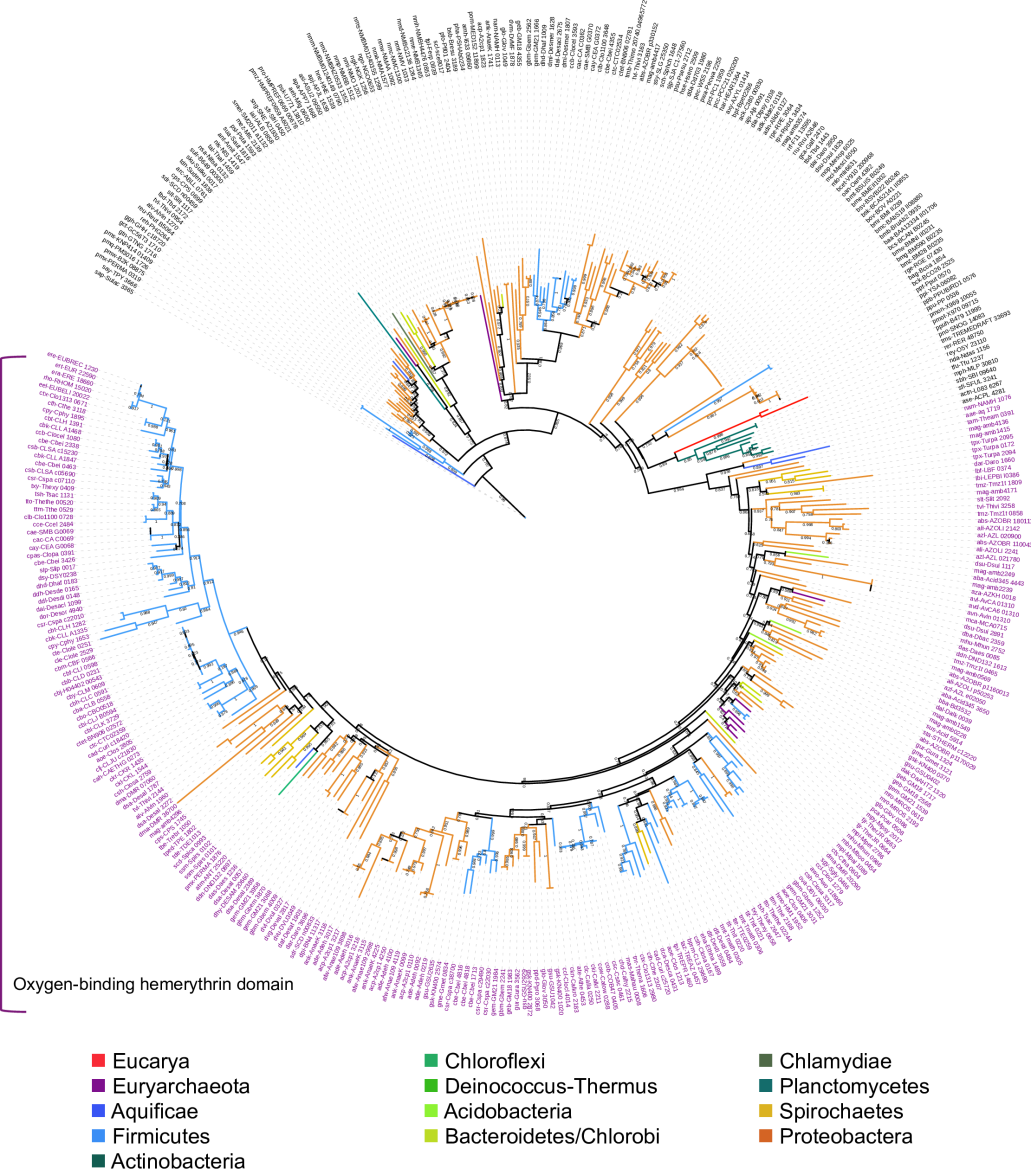
Single-domain hemerythrin/HHE cation-binding domain maximum likelihood tree. Midpoint-rooted maximum-likelihood tree of single domain hemerythrin/HHE cation-binding domain sequences. Internal nodes with approximate likelihood-ratio test lower than 0.6 were collapsed. Each one of the nodes represents a sequence identified by the Pfam-A hemerythrin/HHE cation-binding domain profile in a database of completely sequenced cellular genomes. Sequences names appear at the tips of the branches. Names in purple indicate sequences also identified by a hand-curated oxygen-binding hemerythrin profile.

Oxygen-binding hemerythrin (O_2_-binding Hr) homologs were identified based on the statistical significance of aligning O_2_-binding Hr sequences from two annelid species, *Phascolopsis gouldii* [13] and *Themiste hennahi* [14], with non-mutated protein sequences annotated as hemerythrin or hemerythrin-like proteins in the PDB. The sequences of two O_2_-binding Hrs, from the proteobacteria *Methylococcus capsulatus* [15] and *Desulfovibrio vulgaris* [16], were statistically similar to the annelid sequences (Table 1), and were used as queries in subsequent sequence similarity searches. In contrast, the N-terminal domain of the human F-box and leucine-rich repeat protein 5 (FBXL5), which possess an iron-coordination site that does not bind oxygen [17–19], was not significantly similar to any of the annelid hemerythrin sequences used here as reference (Table 1). We constructed a profile Hidden Markov Model (S1 Fig) with 148 nodes, exclusively based on O_2_-binding Hr homologues, which constitute a divergent monophyletic sub-group of the hemerythrin/HHE (histidine, histidine, glutamic acid) cation-binding domain (Fig 1).

**Table 1:** Statistical significance of the pairwise alignment of hemerythrin and hemerythrin-like sequences with annelid hemerythrins.

Reference sequence (Sequence source)	Subject sequence (Sequence source)	E()	% identity
2MHR (*Themiste hennahi*)	1I4Y (*Phascolopsis gouldii*)	8e-22	45.8
	4XPX (*Methylococcus capsulatus*)	0.00064	27.8
	3AGT, 3AGU, 2AVK, 2AWY (*Desulfovibrio vulgaris*)	0.00019	26.0
	3U9J, 3U9M, 3V5X, 3V5Y, 3V5Z (*Homo sapiens*)	NS	NS
1I4Y (*Phascolopsis gouldii*)	2MHR (*Themiste hennahi*)	3.5e-23	45.8%
	4XPX (*Methylococcus capsulatus*)	0.00045	28.3%
	3AGT, 3AGU, 2AVK, 2AWY (*Desulfovibrio vulgaris*)	0.0058	26.7%
	3U9J, 3U9M, 3V5X, 3V5Y, 3V5Z (*Homo sapiens*)	NS	NS

PDB: Protein Data Bank. E(): Expect value. NS: No sequences with E() < 1000.

### Architecture of hemerythrin-containing sequences

**Fig 2:**
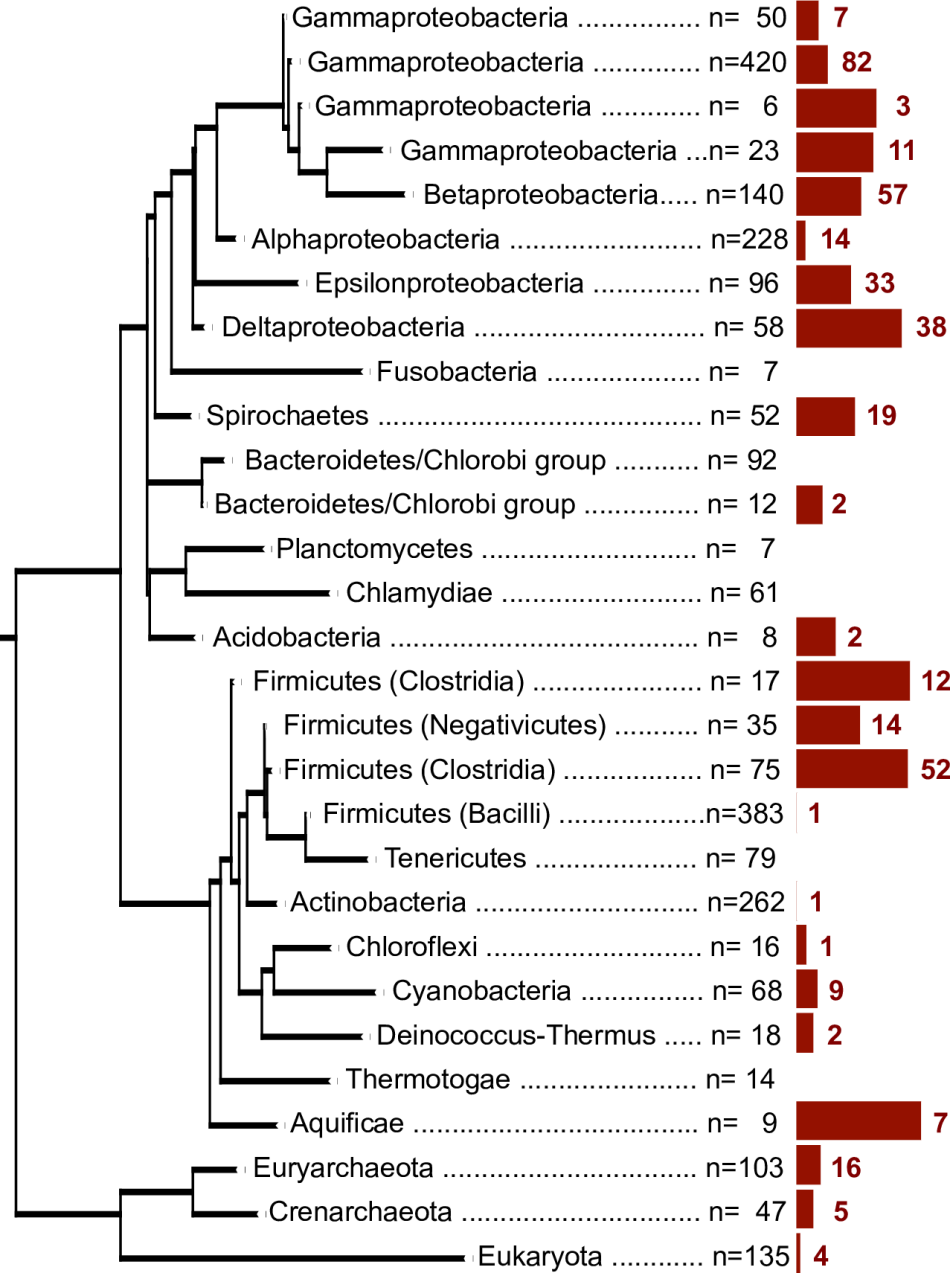
Relative fequency of genomes encoding for hemerythrin domain homologues across a species phylogeny. Phylogenetic tree based on a small subunit rRNA guide tree containing only completely sequenced species. Bacterial and archaeal species are collapsed on the phylum and group level. Eukaryotic species are collapsed together. n: number of species contained within each collapsed branch. The red bar is proportional to the number of species with at least one hemerythrin sequence in each collapsed branch. The total number of genomes with at least one O2-binding hemerytrhin is indicated by a red number next to the bar.

**Fig 3:**
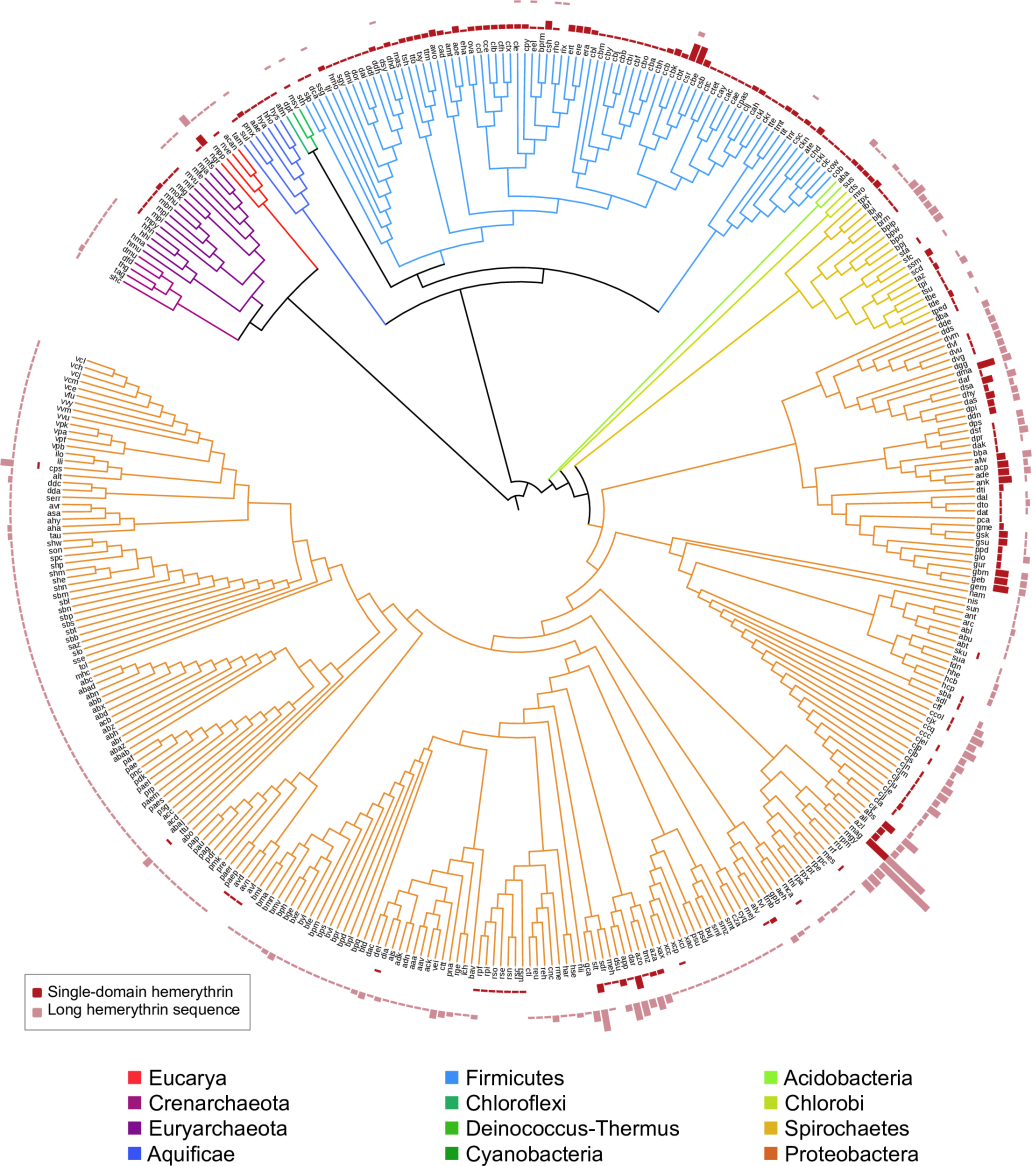
Phylogenetic tree based on species encoding for at least one hemerythrin protein domain. Phylogenetic tree based on a small subunit rRNA guide tree. Branch lengths are arbitrary. Each node corresponds to a completely sequenced species with at least one O_2_-binding Hr sequence homolog. Species names were replaced by their unique KEGG Organisms code [20]. The height of the bar charts is proportional to the absolute number of O_2_-binding Hr copies in each category: single domain O_2_-binding Hr sequences (red), long O_2_-binding Hr sequences (pink).

**Fig 4:**
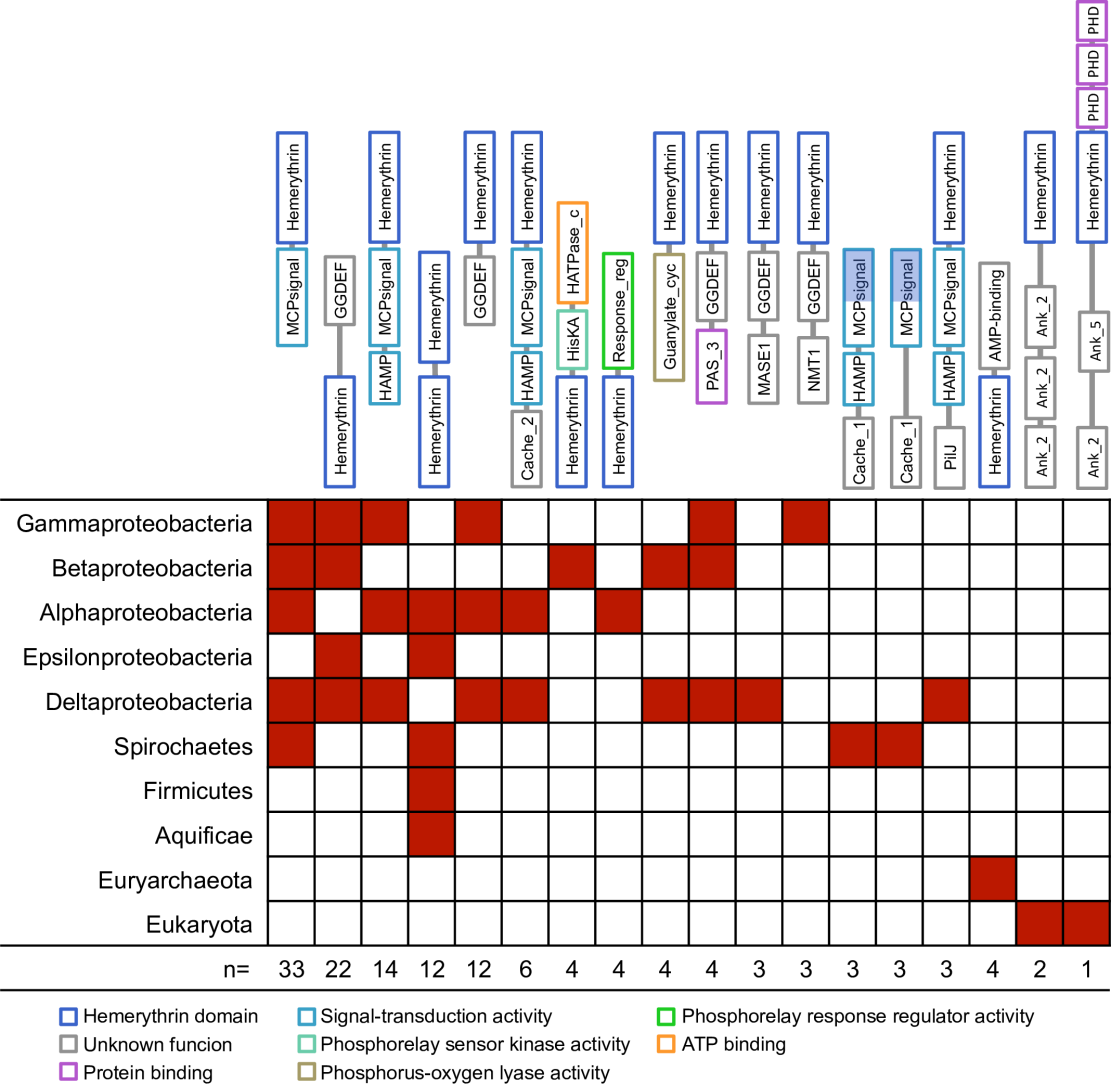
Presence/absence matrix of the different protein domain architectures in bacterial, archaeal and eukaryotic species. Protein domain architectures were obtained as specified in the Methods section. Defined Pfam-A domains are represented by boxes. Only architectures where three or more protein sequences were identified are depicted here. The size of the boxes representing each domian and spacing between contiguous boxes are arbitrary. Hemerythrin domain overlapping other protein domains is represented as half-filled blue boxes.

O_2_-binding Hr sequences were identified in 367/2236 bacterial, 21/150 archaeal and 4/135 eukaryotic genomes (Fig 2). Sequences with subject coverage lower than 85% were considered long sequences and the presence of additional protein domains was investigated using the Pfam-A database (Figs 3 and 4). O_2_-binding Hr homologues were found in the same proportion as single-domain and as long protein sequences in archaeal, bacterial and eukaryotic genomes overall. A total of 56 different domain architectures were identified in long O_2_-binding Hr sequences (Fig 4 and S2 Fig). The largest group of long O_2_-binding Hr sequence architectures (56.9%) contained N-terminal or C-terminal regions of variable size with no clear homologs in Pfam-A. The most frequent location of the O_2_-binding Hr domain in long protein sequences is the protein termini (117 proteins at the C-terminus, 52 proteins at the N-terminus), suggesting a later incorporation by gene fusion events. The most frequent architectures were: 1) N-terminal methyl accepting chemotaxis protein domain (MCP signal) with a C-terminal O_2_-binding Hr domain (33 protein sequences); 2) N-terminal O_2_-binding Hr domain with a C-terminal diguanylate cyclase (GGDEF) domain (22 protein sequences); and 3) N-terminal histidine kinases, adenyl cyclases, methyl-accepting proteins and phosphatases (HAMP) domain with a MCP signal and a C-terminal O_2_-binding Hr domain (14 protein sequences). The Gene Ontology terms associated to the Pfam-A domains identified corresponded to: 1) signal transduction (43%); 2) phosphorelay response regulation (7%); and 3) protein binding (6%).

### Phylogenetic distribution of oxygen-binding hemerythrin sequences

Of the 2521 cellular genomes that were analyzed in this work, a total of 392 (367 bacterial, 21 archaeal and 4 eukaryotic genomes) encoded for O_2_-binding Hr sequences (Fig 2). The number of O_2_-binding Hr copies in a genome varies widely, in particular, species of Spirochaetes, Chlorobi, Acidobacteria, Firmicutes-Clostridia, and α-, β- and δ- Proteobacteria present several copies of single-domain O_2_-binding Hr sequences (Fig 3). The greatest number of single-domain O_2_-binding Hr copies was found in *Magnetospirillum magneticum AMB-1*, a facultative α-Proteobacteria encoding 15 paralogous sequences (Fig 3).

The sample studied here included archaeal genomes only from the Crenarchaeota and Euryarchaeota phyla due to an under-representation of Archaea in genome databases and the low agreement between the databases consulted (See the Methods section). Within Crenarchaeota, O_2_-binding Hr was found only in five species of the order Desulfurococcales within long protein sequences. In Euryarchaeota, five species of the Methanomicrobia class contained single-domain O_2_-binding Hr, while six species of the Methanococci class had either single-domain O_2_-binding Hr or long sequences consisting of an O_2_-binding Hr domain with an AMP-binding domain or with a short orphan elongation. Eukaryotic O_2_-binding Hr sequences were found in *Micromonas pusilla* CCMP1545 and *Acanthamoeba castellanii* Neff only as single-domain sequences, and in *Nematostella vectensis* and *Naegleria gruberi* as both single-domain and long protein sequences. The profile Hidden Markov Model used in this work identified a negligible number of O_2_-binding Hr sequences in eight bacterial phyla (Thermotogae, Actinobacteria, Tenericutes, Chlamydiae, Planctomycetes, Bacteroidetes and Fusobacteria) and in the class Bacilli of Firmicutes (Fig 2). With the exception of Fusobacteria and Tenericutes, the homologous hemerythrin/HHE cation-binding domain was found in species from the phylogenetic groups where O_2_-binding Hr was not present (S3 Fig). In early-branching Bacteria (Aquificae, Chloroflexi, Deinococcus-Thermus and Cyanobacteria) and Firmicutes, single-domain O_2_-binding Hr accounted for 91.6% of the sequences identified. In contrast, in later bacterial groups (Acidobacteria, Chlorobi, Spirochaetes, and Proteobacteria) only 31.9% of the O_2_-binding Hr homologues were single-domain sequences.

As shown in Fig 2, the sequences of single-domain HHE cation-binding hemerythrin and single-domain O_2_-binding Hr, which we have analyzed in this work, are the outcome of an ancient gene duplication predating the explosive divergence of Bacteria during Precambrian times. The maximum-likelihood tree in Fig 2 also shows that the single-domain O_2_-binding Hr is a well-defined, monophyletic, highly divergent group distinct from the hemerytrin/HHE cation-binding domain.

**Fig 5:**
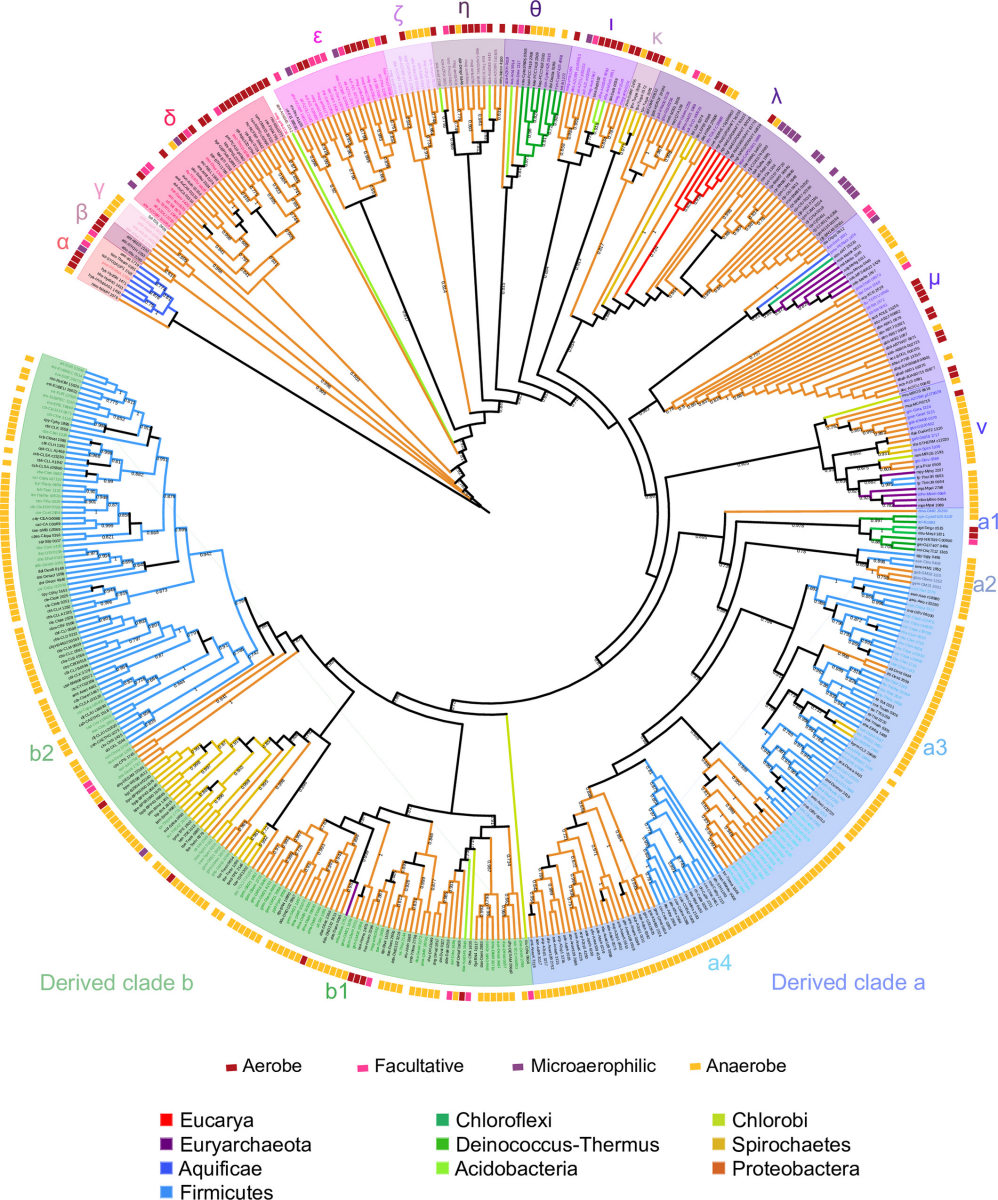
Maximum-likelihood tree of single-domain hemerythrin sequences. Internal nodes with approximate likelihood-ratio test lower than 0.6 were collapsed. Names of the sequences appear at the tips of the branches. Sub-clusters are named by Greek letters on the base of the tree and by letters and numbers in the direved clusters. Node names appear in color when there is at least another copy of single-domain O_2_-binding Hr in a separate cluster of the tree. Oxygen requirement of the species source, according to the Genomes OnLine Database [21], is indicated by color bars at the tips of the nodes.

The tree in Fig 5 shows two well-defined single-domain O2-binding Hr derived groups, a and b, formed by sequences encoded mostly by anaerobic species. The derived clade a includes sequences from Deinococcus-Thermus; Cyanobacteria; Firmicutes; Spirochaetes; and δ-Proteobacteria. The derived clade b is formed by sequences from Acidobacteria; Chlorobi; α-, β-, γ- and δ-Proteobacteria, as well as from the archaeal *Methanospirillum hungatei* JF-1, where it is present most likely because of a lateral gene event. Sequences in both derived clusters diverged from an early gene duplication of the single-domain O_2_-binding Hr gene, and also appear to have been subject of lateral gene transfer, gene loss, gene duplication and orthologous replacement events.

There are several cases in which more than one O_2_-binding Hr sequence can be identified in a single cellular genome. This may be due to lateral gene transfer events or, most likely, to paralogous duplication events (Table 2).

**Table 2:** Species with more than one genomic copy of single-domain O2-binding Hr.

	Species name	n_c_
Bacteria		
α-Proteobacteria	*Magnetospirillum magneticum* AMB-1	15
	*Azospirillum brasilense* Sp245	8
	*Azospirillum lipoferum* 4B	4
	*Azospirillum* sp. B510	3
β-Proteobacteria	*Dechloromonas aromatica* RCB	7
	*Sideroxydans lithotrophicus* ES-1	6
	*Thauera* sp. MZ1T	4
	Candidatus *Accumulibacter phosphatis* clade IIA UW-1	3
	*Dechlorosoma suillum* PS	3
	*Azoarcus* sp. KH32C	2
	*Azoarcus* sp. BH72	2
	*Herbaspirillum seropedicae* SmR1	2
	*Sulfuricella denitrificans* skB26	2
γ-Proteobacteria	*Teredinibacter turnerae* T7901	3
	*Thiocystis violascens* DSM 198	2
δ-Proteobacteria	*Desulfovibrio magneticus* RS-1	9
	*Geobacter* sp. M21	9
	*Geobacter bemidjiensis* Bem	8
	*Anaeromyxobacter dehalogenans* 2CP-C	8
	*Anaeromyxobacter dehalogenans* 2CP-1	7
	*Anaeromyxobacter* sp. Fw109-5	7
	*Anaeromyxobacter* sp. K	7
	*Geobacter* sp. M18	7
	*Desulfovibrio salexigens* DSM 2638	6
	*Geobacter sulfurreducens* KN400	5
	*Geobacter sulfurreducens* PCA	5
	*Desulfovibrio aespoeensis* Aspo-2	4
	*Desulfovibrio desulfuricans* ND132	3
	*Desulfovibrio piezophilus* C1TLV30	3
	*Geobacter lovleyi* SZ	3
	*Desulfobacterium autotrophicum* HRM2	2
	*Desulfovibrio hydrothermalis* AM13 = DSM 14728	2
	*Desulfomonile tiedjei* DSM 6799	2
	*Desulfobacula toluolica* Tol2	2
	*Geobacter metallireducens* GS-15	2
	*Geobacter uraniireducens* Rf4	2
	*Pelobacter propionicus* DSM 2379	2
ε-Proteobacteria	*Campylobacter jejuni* 4031	3
	*Campylobacter jejuni* subsp. jejuni 81–176	2
	*Campylobacter jejuni* subsp. jejuni M1	2
	*Campylobacter jejuni* subsp. jejuni 81116	2
Spirochaetes	*Spirochaeta smaragdinae* DSM 11293	3
	*Treponema brennaborense* DSM 12168	3
	*Turneriella parva* DSM 21527	3
	*Brachyspira pilosicoli* P43/6/78	2
	*Brachyspira pilosicoli* 95/1000	2
	*Brachyspira murdochii* DSM 12563	2
	*Treponema azotonutricium* ZAS-9	2
	*Treponema denticola* ATCC 35405	2
	*Treponema pedis* T A4	2
	*Treponema primitia* ZAS-2	2
Bacteroidetes/Chlorobi	*Chloroherpeton thalassium* ATCC 35110	2
	*Melioribacter roseus* P3M-2	2
Acidobacteria	Candidatus *Koribacter versatilis* Ellin345	5
	Candidatus *Solibacter usitatus* Ellin6076	2
Firmicutes	*Clostridium beijerinckii* NCIMB 8052	8
	*Clostridium saccharoperbutylacetonicum* N1-4(HMT)	8
	*Clostridium saccharolyticum* WM1	4
	*Alkaliphilus oremlandii* OhILAs	3
	*Caldicellulosiruptor bescii* DSM 6725	3
	*Acetobacterium woodii* DSM 1030	3
	*Clostridium botulinum* B Eklund 17B (NRP)	3
	*Clostridium lentocellum* DSM 5427	3
	*Clostridium saccharobutylicum* DSM 13864	3
	*Agathobacter rectalis* M104/1	3
	*Agathobacter rectalis* ATCC 33656	3
	*Agathobacter rectalis* DSM 17629	3
	*Clostridium acidurici* 9a	2
	*Clostridium autoethanogenum* DSM 10061	2
	*Clostridium botulinum* A3 Loch Maree	2
	*Clostridium botulinum* E3 Alaska E43	2
	*Clostridium cellulovorans* 743B	2
	*Clostridium cellulolyticum* H10	2
	*Clostridium clariflavum* DSM 19732	2
	*Clostridium* sp. BNL1100	2
	*Clostridium ljungdahlii* DSM 13528	2
	*Lachnoclostridium phytofermentans* ISDg	2
	*Caldicellulosiruptor saccharolyticus* DSM 8903	2
	*Ruminiclostridium thermocellum* ATCC 27405	2
	*Ruminiclostridium thermocellum* DSM 1313	2
	*Desulfitobacterium dehalogenans* ATCC 51507	2
	*Desulfitobacterium hafniense* DCB-2	2
	*Desulfitobacterium hafniense* Y51	2
	*Oscillibacter valericigenes* Sjm18-20	2
	*Thermoanaerobacter italicus* Ab9	2
	*Thermincola potens* JR	2
	*Thermoanaerobacter mathranii* subsp. mathranii A3	2
	*Thermoanaerobacterium saccharolyticum* JW/SL-YS485	2
	*Thermoanaerobacterium thermosaccharolyticum* DSM 571	2
	*Thermoanaerobacterium thermosaccharolyticum* M0795	2
	*Thermoanaerobacterium xylanolyticum* LX-11	2
Cyanobacteria	*Cyanothece* sp. PCC 7425	3
	*Halothece* sp. PCC 7418	3
	*Thermosynechococcus elongatus* BP-1	2
Aquificae	*Persephonella marina* EX-H1	2
**Archaea**		
Euryarchaeota	*Methanospirillum hungatei* JF-1	2
**Eucarya**		
	*Naegleria gruberi*	5

nc: Number of genomic copies of single domain O_2_-binding Hr.

The many cases in which highly similar single-domain O_2_-binding Hr copies are closely grouped in the same cluster in the phylogenetic tree suggest recent paralogous duplication events. For instance, *Magnetospirillum magneticum* AMB-1, a facultative proteobacteria, encodes for 15 single-domain O_2_-binding Hr gene copies, some of which are located in the same cluster, while others are found in distant clusters: amb4136 is represented by a single branch; amb4265, amb2654, amb4171, amb2239 and amb1415 are in cluster ε; amb1987, amb0226, amb1549 and amb2249, in cluster ι; amb3418, amb4296 and amb3966 in cluster η; amb0569 in cluster ζ; and amb1952 in cluster b1, indicating duplication events that occurred at different times during evolution of *M*. *magneticum* AMB-1 (Fig 5). Alternatively, the peculiarities of the distribution of one or more O_2_-binding Hr gene copies can be explained by lateral gene transfer events.

The overall topology of the single-domain O_2_-binding Hr phylogenetic tree is in clear disagreement with the 16/18S rRNA reference tree (Fig 5). This indicates that the significance of O_2_-binding Hr as a phylogenetic marker is hindered by a complex history of gene losses, gene duplications, paralogous replacements and lateral gene transfer events. The base of the tree is characterized by a highly branching pattern of single-domain O_2_-binding Hr sequences encoded by aerobic organisms including Bacteria, Archaea and Eukarya (Fig 5 and Table 3). The ample distribution of highly divergent O_2_-binding Hr sequences among aerobic organisms is probably best understood by the adaptive value of oxygen-binding proteins in a Precambrian environment that was becoming increasingly oxidizing as time went by.

**Table 3:** Name of the protein sequences at each sub-group of the phylogenetic tree of single-domain O2-binding Hr.

Sub-group name	Protein name	Phylogenetic group of the sequence source
α	NAMH_1076	Epsilonproteobacteria
	HY04AAS1_1450, HydHO_1431, HydSN_1471, PERMA_1769, SYO3AOP1_1768, Theam_0391, aq_1719	Aquificae
β	Abu_2290, A7H1H_2253, ABED_2102	ε-Proteobacteria
γ	Anae109_3052, A2cp1_0412, Adeh_0383, AnaeK_0411	δ-Proteobacteria
	TOL_2403	γ-Proteobacteria
δ	AZOBR_100054, AZOLI_2220	α-Proteobacteria
	Slit_0876, azo0535, Daro_1420, Dsui_1348, BTH_I1789, BTI_1258, GBP346_A2891, BPSL2377, Tmz1t_0248, Rpic12D_0794, Rpic_0724, RCFBP_20642, RSPO_c02590, CMR15_30122, F504_795, RSc0777	β-Proteobacteria
	TERTU_1569, TERTU_0429, AvCA_01310, AvCA6_01310, Avin_01310, PCA10_p0360	γ-Proteobacteria
	Sulku_1024, Arnit_0274, ABLL_1168	ε-Proteobacteria
	amb4136	α-Proteobacteria
	Acid345_2152	Acidobacteria
ε	F11_08530, Rru_A1655, AZOBR_180111, AZOBR_110043, AZOLI_2142, AZOLI_2241, AZL_020900, AZL_021780, amb1415, amb4171, amb4265, amb2239, amb2654	α-Proteobacteria
	Daro_2268, Daro_1406, Daro_1670, Slit_2092, Tmz1t_0858	β-Proteobacteria
	Thivi_3258	γ-Proteobacteria
ζ	amb0569, AZOBR_70025, AZOBR_p140089	α-Proteobacteria
	CAP2UW1_1286	β-Proteobacteria
	Anae109_0548, Adeh_0492, A2cp1_0520, AnaeK_0525	δ-Proteobacteria
η	amb3418, amb3966, amb4296, AZOBR_140105, Meso_4300	α-Proteobacteria
	AZKH 0401, Tmz1t 0465, Tmz1t 1809, CAP2UW1_3698	β-Proteobacteria
	Despr_0446	δ-Proteobacteria
	Acid_2398, Acid345_4443	Acidobacteria
θ	AZKH_0018, Dsui_1117	β-Proteobacteria
	Acid_5914	Acidobacteria
	Cyan10605_0305, PCC7418_2308, PCC7418_2309, PCC7418_2310, Cyan7425_2835, Cyan7425_4668, Dacsa_0199, tlr1372	Cyanobacteria
ι	amb2249, amb1987, amb1549, amb0226, AZOBR_p1160013, AZOLI_p50253, AZL_e02050	α-Proteobacteria
	Bd3532, Dalk_0039	δ-Proteobacteria
	Acid345_3650, Acid345_3651	Acidobacteria
κ	DvMF_2455	δ-Proteobacteria
	Turpa_2094, Turpa_0172, Turpa_2095	Spirochaetes
λ	GSU0256, M301_1805, SCD_n00023, CAP2UW1_3898	β-Proteobacteria
	HDN1F_19180, Q7A_109	γ-Proteobacteria
	UWK_00512, KN400_0228, GSU0256, Gbem_2255, GM21_1969, HRM2_20500, HRM2_34410, TOL2_C18880	δ-Proteobacteria
	CJJ81176_0083, Sulba_1991, Sdel_1887, Cla_1104, G157_00720, BN867_00640, BN867_09640, BN867_02160, CJM1_0949, CJM1_0224, C8J_0913, C8J_0219, BN865_01820, A911_01165, CJSA_0218, Cj0241c, CJJ81176_0266, N135_00164, BN148_0241c	ε-Proteobacteria
	LBF_0374, LEPBI_I0386	Spirochaetes
	NEMVE_v1g100902, NAEGRDRAFT_36233, NAEGRDRAFT_83311, NAEGRDRAFT_83016, NAEGRDRAFT_81770, NAEGRDRAFT_44819	Eucarya
μ	Dtpsy_0612, Dsui_2891, Daro_1660, Slit_2372, Slit_0061	β-Proteobacteria
	XCR_2629, TERTU_0969, AOLE_15255, ABZJ_00882, ABK1_0879, ABTJ_02921, AB57_0939, M3Q_1087, ABTW07_0871, ABBFA_002723, BDGL_000155, P795_13310, BJAB0868_00901, ABD1_08370, BJAB0715_00877, A1S_0891, ACICU_00842	γ-Proteobacteria
	DMR_08570	δ-Proteobacteria
	ANT_25220	Chloroflexi
	PERMA_1876	Aquificae
	Metok_0621, Metin_1245, Metig_0111, Metvu_0446, MFS40622_1429, Mefer_1067	Euryarchaeota
ν	AZOBR_p1170029	α-Proteobacteria
	MCA0715	γ-Proteobacteria
	Gura_1324, Gmet_3121, KN400_0370, GSU0402, DaAHT2_1320, GM18_1717, Glov_0386, Pcar_0508	δ-Proteobacteria
	STHERM_c12220, Spirs_1036	Spirochaetes
	MROS_0616, MROS_2193	Bacteroidetes/Chlorobi
	TherJR_0663, TherJR_0664	Firmicutes
	Mpsy_2017, Mpet_2786, Mhun_0966, Mboo_0454, Mpal_1089	Euryarchaeota
Derived clade a		
	DMR_20290	δ-Proteobacteria
a1	Cyan7425_5247, tlr1993, NIES39_C00800, GEI7407_0496, Osc7112_1305	Cyanobacteria
	Deipr_0515, Mesil_1871	Deinococcus-Thermus
a2	GM18_1110, Gbem_1252, GM21_3031	δ-Proteobacteria
	Sgly_0466, Clos_0406, HM1_1952	Firmicutes
a3	Desti_0484, Desti_3559	δ-Proteobacteria
	TREAZ 0457, TREPR_1460	Spirochaetes
	Awo_c18980, Awo_c20290, Awo_c32720, CL3_29690, Cbei_1713, Cbei_2165, Cbei_3755, Cbei_4816, Cbei_4818, Clo1313_2980, Clocl_1279, Clos_1213, Closa_0167, Closa_2585, Closa_3317, Cspa_c22030, Cspa_c22470, Cspa_c29490, Cspa_c38700, Cspa_c44550, Cthe_2307, Curi_c25720, DSY1174, Desca_0431, Desde_1795, Desmer_2419, Dhaf_2262, ERE_18800, EUBREC_1216, EUR_17090, Ethha_1489, OBV_06010, OBV_06030, TTE0259, Thethe_02244, Thexy_0658, Thit_0220, Thit_0221, Tmath_0305, Tmath_0306, Tsac_2647, Tthe_2168	Firmicutes
a4	2cp1_3216, A2cp1_0110, A2cp1_0602, A2cp1_3217, A2cp1_4250, Adeh_0092, Adeh_0219, Adeh_0575, Adeh_3016, Adeh_3017, Adeh_4100, Anae109_0618, Anae109_2752, Anae109_2998, Anae109_3898, Anae109_4119, AnaeK_0099, AnaeK_0610, AnaeK_3115, AnaeK_3116, AnaeK_4225, GM18_1967, GM18_1983, GM21_1962, GM21_1984, GSU1042, GSU2635, GSU2929, Gbem_2241, Gbem_2262, Glov_1974, Glov_3050, Gmet_0834, Gura_3562, KN400_1020, KN400_2574, KN400_2872, Ppro_3068	δ-Proteobacteria
	Athe_0453, Athe_2564, Athe_2568, COB47_0405, Calhy_2215, Calkr_2211, Calkro_2183, Calla_0250, Calow_0288, Ccel_0173, Clo1100_0178, Clocl_4014, Csac_0285, Csac_0461, Mahau_0008, STH1393, Thena_1606	Firmicutes
Derived clade b		
	Ctha_0604	Bacteroidetes/Chlorobi
b1	amb1952	α-Proteobacteria
	SCD_n00833, Daro_3696, Daro_2683, Hsero_2396, Hsero_2403, Slit_1555,	β-Proteobacteria
	Thimo_2708, Alvin_1960, Thivi_2144	γ-Proteobacteria
	BN4_10860, BN4_11024, BN4_11317, DESAM_20660, DMR_07060, DMR_29930, DMR_30270, DMR_30500, DMR_36700, DMR_36710, DMR_41660, DND132_0897, DND132_1613, DND132_3219, DVU3049, Daes_0085, Daes_2600, Daes_2883, Dbac_2359, Dde_0253, Desaf_1903, Desal_0057, Desal_2389, Desal_3272, Desal_3481, Deval_2817, Dvul_0327, GM18_2568, GM18_2640, GM21_1467, GM21_1467, GM21_1539, GM21_3068, GM21_4102, Gbem_2701, Gbem_2773, Gbem_4009, Ppro_2058	δ-Proteobacteria
	Ctha_1635	Bacteroidetes/Chlorobi
	Acid345_3649	Acidobacteria
	Mhun_2752	Euryarchaeota
b2	Slit_2549, azo3759	β-Proteobacteria
	CPS_1745	γ-Proteobacteria
	TOL2_C41160, GM18_4299, Gbem_3870, GM21_3958, DESAM_22185, Desal_1787, Desal_1268	δ-Proteobacteria
	B2904_orf2240, BP951000_1675, BP951000_1676, BPP43_06840, BPP43_06845, Bint_2815, Bmur_0987, Bmur_1401, Spica_0993, TDE1013, TDE1302, TPE_1802, TPE_2146, TREAZ_2611, TREPR_1778, Trebr_0879, Trebr_0880, Trebr_1050, Tresu_0834, WESB_0512	Spirochaetes
	Amet_4661, BN906_02572, CA_C0069, CAETHG_0273, CAETHG_1518, CBF_0566, CBO0518, CEA_G0068, CKL_1544, CKR_1435, CLB_0558, CLC_0591, CLD_0231, CLH_1282, CLH_1391, CLI_0598, CLJ_B0594, CLJU_c21830, CLJU_c36090, CLK_1558, CLK_3729, CLL_A1335, CLL_A1468, CLL_A1847, CLM_0609, CLSA_c05690, CLSA_c15230, CLSA_c33120, CTC02359, Cbei_0463, Cbei_2338, Cbei_3426, Ccel_2484, Clo1100_0728, Clo1313_0671, Clocel_1080, Clocel_3467, Clole_0251, Clole_2025, Clole_2529, Clopa_0391, Clos_2805, Closa_2759, Cphy_1653, Cphy_1895, Cspa_c07110, Cspa_c22010, Cspa_c46400, Cthe_3118, Curi_c18420, DSY0238, Desaci_1099, Desde_0165, Desdi_0148, Desor_4940, Dhaf_0183, ERE_18660, ERE_24470, EUBELI_20022, EUBREC_0514, EUBREC_1230, EUR_01540, EUR_22590, H04402_00543, RHOM_15020, SMB_G0069, Slip_0017, Thethe_00520, Thexy_0409, Tsac_1131, Tthe_0529	Firmicutes

Agreement with the topology of the species tree was observed mainly at the sub-group level of the O_2_-binding Hr sequence tree. For instance, as shown in Fig 5 and Table 3, sequences from Aquificae form a minor sub-group (sub-group α), suggesting that a single-domain O_2_-binding hemerythrin was present in their common ancestor. A comparable situation may be seen in the θ sub-group, which can be interpreted as the outcome of vertically inherited single-domain O_2_-binding Hr in species of Cyanobacteria subclass Oscillatoriophycideae. In the case of archaeal sequences, Methanococci and Methanomicrobia form two separate groups. Sequences from Methanococci, and bacterial sequences from *Anaerolinea thermophila* UNI-1, *Persephonella marina* EX-H1, *Dechlorosoma suillum* PS and *Acidovorax ebreus* TPSY form cluster μ. A distinct independent sub-group ν is formed by sequences from Methanomicrobia, and bacterial sequences from *Melioribacter roseus* P3M-2, *Geobacter lovleyi* SZ and *Pelobacter carbinolicus* DSM 2380. The topology of the single domain O_2_-binding Hr tree, together with the absence of single-domain O_2_-binding Hr in most archaeal groups, could indicate the acquisition by Methanomicrobia and Methanococci of the single-domain O_2_-binding Hr sequences in two independent events of lateral gene transfer. Eukaryotic sequences from *Nematostella vectensis* and *Naegleria gruberi* cluster together in sub-group λ.

## Discussion

The HHE cation-binding domain was first predicted by bioinformatics methods as a domain composed of two helical regions and a conserved HHE cation-binding site [22]. The hemerythrin-like domain family is a repetition of the HHE cation-binding domain, which folds into an up-and-down bundle of four left-handed helices [14,23]. The molecular function of proteins containing the hemerythrin/HHE cation-binding domain, including metazoan oxygen-carrier hemerythrins, is often related to O_2_ or reactive oxygen species responses [10,16,24–29].

The search for O_2_-binding Hr domain homologs was performed using a manually curated profile Hidden Markov Model, and resulted in the identification of sequences of 86 to 2425 amino acids length, using a profile that contained 148 nodes (S1 Fig), highly weighting the position of the iron-coordinating amino acids in X-ray solved structures of O_2_-binding Hrs. Sequences with subject coverage lower than 85% were considered long sequences. To identify the presence of possible additional domains, we searched the protein profile database Pfam-A, which confirmed known additional domains in 37.2% of long sequences. In agreement with previous bioinformatics searches [9,11], the most frequent domain found in long O_2_-binding Hr sequences was the MCP signal domain (Fig 4). The characterization of the hemerythrin domain of the methyl-accepting chemotaxis protein dcrH from *Desulfovibrio vulgaris* has shown that the four-helix bundle fold and the amino acids of the active site are conserved [24]. It has been proposed that the hemerythrin domain in this structure could have a role in signal transduction [30].

O_2_-binding Hr sequences were identified in two archaeal groups (Euryarchaeota and Crenarchaeota), ten bacterial groups (Aquificae, Deinococcus-Thermus, Cyanobacteria, Chloroflexi, Firmicutes, Acidobacteria, Chlorobi, Spirochaetes and Proteobacteria), and four eukaryotic species (*Naegleria gruberi*, *Micromonas pusilla CCMP1545*, *Nematostella vectensis* and *Acanthamoeba castellanii*). It is also known to be present in marine invertebrates (Sipuncula, Brachiopoda, Priapulida and Annelida) [8]. By far, the highest number of O_2_-binding Hr sequences is found in Proteobacteria (Figs 2 and 3).

Single-domain and long O_2_-binding Hr-containing sequences are not randomly distributed in the phylogenetic tree, but exhibit a differential distribution among taxonomic groups, particularly in Bacteria, where two separate groups can be distinguished. In the first bacterial group, that includes deep-branching bacteria and the Firmicutes-Clostridia clade, O_2_-binding Hr homologues are predominantly single-domain sequences (91.6%). The expression of the single-domain HerA hemerythrin in the microaerophilic bacteria *Campylobacter jejuni* [26] reduces its susceptibility to oxygen and hydrogen peroxide-mediated damage of two iron-sulphur cluster enzymes (pyruvate:acceptor oxydoreductase and 2-oxoglutarate:acceptor oxidoreductase). This suggest that single-domain O_2_-binding Hr may also be involved in the prevention of oxygen-mediated damage in microaerophilic aquificales and in anaerobic clostridia, and probably reflect an evolutionary adaptation to the presence of free molecular oxygen. The absence of O_2_-binding Hr homologues in Mollicutes and the class Bacilli of Firmicutes, two bacterial groups closely related to clostridial species, is probably due to secondary loss.

The second bacterial group includes Acidobacteria, Chlorobi, Spirochaetes and Proteobacteria. Within this group, long O_2_-binding Hr sequences (68.1%) are more frequent than single-domain (31.9%) O_2_-binding Hr. In Proteobacteria, which is the most diverse bacterial phylum, the study of the O_2_-binding Hr domain led to the identification of three different functions: a) as a 135-amino acid domain in a chemotactic protein of *Desulfovubrio vulgaris* [24]; b) as a single-domain protein McHr in *Methylococcus capsulatus*, that supplies oxygen to the membrane-bound methane monooxygenase [10]; and c) as a single-domain protein HerA in *Campylobacter jejuni*, which protects iron-sulphur cluster enzymes from oxidative damage [26]. This exemplifies how the O_2_-binding Hr domain may have been coopted into specific physiological functions by different species, particularly in later-branching bacteria, where it was incorporated into a wide variety of sequences. In some cases, the additional sequences within long O_2_-binding Hr sequences are group-specific. For instance, long O_2_-binding Hr sequences from Acidobacteria and Chlorobi exhibit elongations that do not match domains in the Pfam-A database, and two architectures of the long O_2_-binding Hr sequences from Spirochaetes genomes contain a domain called Cache_1, which is an acronym for calcium channels and chemotaxis receptor.

Variations at the iron-coordinating amino acid residues in 106 single-domain O_2_-binding Hr sequences were observed (S4 Fig). This is quite evident in sequences from the Deinococcus-Thermus, Cyanobacteria, Firmicutes-Clostridia and from the α-, β- and δ- Proteobacteria clades. Amino acid substitutions may modify the reversible oxygen-binding ability of hemerythrins [31], or even the native metal-ion preference. Molecular characterization of the variants could clarify whether sequence variations at the iron-coordination site produce loss of function, neo-functionalization, or alternative iron-binding mechanisms.

Hemerythrin homologues have an ample biological distribution, and are present in the three domains of life. However, as shown here, their distribution is not universal, which is consistent with previous genomic searches, and may be explained by frequent gene losses [11,12]. Although the topology of the O_2_-binding Hr tree indicates intense horizontal gene transfers, gene duplications and differential gene loss (Fig 5), it is clear that O_2_-binding Hr is widely distributed in Clostridia and all five classes of Proteobacteria, suggesting that it is an ancient Precambrian trait that was already present before the divergence of Firmicutes and Proteobacteria. Alternatively, the evolution of O2-binding Hr may have occurred in a divergent phylogenetic group, and was horizontally transferred, through independent events, to the individual orders of Clostridia and/or Proteobacteria. This alternative scenario represents a less parsimonious explanation to the overall O2-binding Hr distribution. Instead, horizontal gene transfer could explain the cases where the presence of O2-binding Hr is circumscribed to a particular family or genus, as appears to be the case for archaean O2-binding Hr.

## Conclusion

The four known evolutionary unrelated oxygen-carrier protein families (hemoglobin, hemerytrhin and the two non-homologous molluscan and arthropodan hemocyanins) represent a polyphyletic response to the selective pressure imposed by the accumulation of free oxygen during the Precambrian. Like other molecular mechanisms involved in the protection and repair of oxygen-induced damage that evolved during Precambrian times [32], the ample biological distribution of O_2_-binding Hr probably reflects its adaptive significance in an environment became increasingly oxidizing. The biological group where hemerythrin first evolved remains unknown, but the distribution of O_2_-binding Hr sequences suggest that its capacity to reversibly bind oxygen was exploited first by prokaryotic species to fulfill a range of different needs, ranging from protection against oxidative damage to oxygen supply to particular enzymes and pathways. More specifically, the available data suggests that it may have originated prior to the divergence of Firmicutes and Proteobacteria. Thereafter, the incorporation of the O_2_-binding Hr domain into preexisting proteins, combined with other mechanisms involved in the protection against oxidative damage, may have allowed a functional diversification of the protein repertoire particularly in the proteobacteria, one of the most diverse groups of bacteria. The evolution of O_2_-binding Hr sequences appears to parallel the evolution of strategies allowing the incorporation of oxygen to biological processes as a consequence of accumulation of free oxygen in the Precambrian oceans and atmosphere.

## Methods

### Sequence similarity searches

Eleven non-mutated hemerythrin and hemerythrin-like amino acid sequences retrieved from the Protein Data Bank (PDB) database [33] were compared to the hemerythrin sequence from *Themiste hennahi* (PDB codes 2MHR) and from *Phascolopsis gouldii* (PDB code 1I4Y) with the program PRSS version 36.3.8c (number of shuffles: 200; scoring matrix: Blosum50; open gap penalty: -10; extension gap penalty: -2) from UVa FASTA Server [34,35]. Expect value lower than 1e-3 of one-to-one alignments was used as cutoff for homologous sequences.

An initial BLAST search against the Kegg Genes database release 75.1[20,36] identified 365 protein sequences with expect value < 1e-5 and subject coverage > 95%. The sequences were aligned with MAFFT G-INS-I algorithm [37] with default parameters (gap opening penalty: 1.53; offset: 0.0). A profile Hidden Markov Model (pHMM) was constructed with HMMER 3.1b1 [38]. The Pfam hemerythrin profile (http://pfam.xfam.org/family/hemerythrin) [39] has a low restriction to include sequences with changes at the iron coordination amino acids observed in oxygen-binding hemerythrins (S1 Fig). The distribution of the hemerythrin domain homologues found by the Pfam profile is shown in S3 Fig. The hand curated hemerythrin pHMM was scanned against 2521 genomes from the Kegg Genes database release 75.1 that were unequivocally traced to an entry of the SSU RefNR99 guide tree from the SILVA SSU database release 119 [40] based on taxonomy identifiers from both databases.

### Domain annotation and associated biological processes

Each sequence was scanned with hmmscan from the HMMER 3.1b1 software using the database of protein families Pfam-A version 28.0 [39]. The hemerythrin profile in Pfam-A was substituted with the pHMM that we generated. Domain overlapping was solved with a pearl script preferring domains with lower expect values. Gene Ontology information on molecular function of Pfam domains was obtained from the Pfam web page [41].

### Phylogenetic analysis

The maximum-likelihood (ML) tree of single-domain oxygen-binding hemerythrin sequences and of hemerythrin HHE cation-binding domain sequences were constructed with PHYML [42]. The parameters for the construction of the trees (Table 4) were automatically selected with SMS: Smart Model Selection using the Akaike Information Criterion at the South of France bioinformatics platform (http://www.atgc-montpellier.fr). Branch support was given by the approximate likelihood-ratio test (aLRT) for branches [43]. Internal nodes with low support (< 0.6) were collapsed. Additional bootstrap support is provided by a 100-replicates as shown in S5 Fig. The multiple sequence alignments were constructed with MAFFT, L-INS-i algorithm using the default parameters (gap opening penalty: 1.53; offset: 0.0). The maximum-likelihood tree of hemerythrin HHE cation-binding domain sequences was midpoint-rooted; the maximum-likelihood of single-domain oxygen-binding hemerythrin sequences was rooted following the topology of the hemerythrin HHE cation-binding domain tree. Tree visualization was made with Interactive Tree Of Life V1.0 [44].

**Table 4:** Model selection and distribution parameters used in the ML trees inference.

	ML tree of HHE cation-binding domain sequences	ML tree of oxygen-binding hemerythrin sequences
Tree topology search	Best of NNIs and SPRs	Best of NNIs and SPRs
Model of amino acids substitution	LG	LG
Number of taxa	389	472
Discrete gamma model		
Number of categories	6	6
Gamma shape parameter	1.224	1.151

## Supporting Information

S1 Fig Conservation of the iron-coordinating amino acids is reflected on the calculated profile Hidden Markov Model. Logo representation of the multiple sequence alignment used as seed to calculate the profile Hidden Markov models. The vertical axis indicates the information content of a sequence position. The one-letter notation for amino acid sequences was used. Glutamic and aspartic acid (purple), histidine (cyan). (A) O_2_-binding Hr model. Positions of the iron-coordinating amino acids are indicated by arrows. Position and length of helical structures were predicted by Ali2D. (B) Pfam-A hemerythrin model.
(TIF)

S2 Fig Schematic reperesentation of the domain architectures of long O_2_-binding hemerythrin sequences. Protein domains identified in long O2-binding hemerythrin sequences are designated by their short Pfam-A family name. The number of sequences showing a particular architecture is indicated after a tabular space.
(TIF)

S3 Fig Proportion of genomes with hemerythrin HHE cation-binding domain. Phylogenetic tree based on a small subunit rRNA guide tree containing only completely sequenced species. Bacterial and archaeal species are collapsed on the phylum level. Eukaryotic species are collapsed together. n: number of species within the collapsed branch. The red bar is proportional to the number of species with at least one hemerythrin HHE cation-binding domain sequence in each group. The total number of genomes with at least one HHE cation-binding domain sequence is indicated by a purple number next to the bar.
(TIF)

S4 Fig Number of single-domain hemerythrin copies with modifications of the iron-coordinating amino acids within each phylogenetic group. (TIF)

S5 Fig Hemerythrin /HHE cation.binding phylogeny (100 bootstrap replicates). Sequences names are the same as in Fig 1.
(TIF)

## Abbreviations

aLRT: approximate likelihood-ratio test
AMP: Adenosine monophosphate
FBXL5: F-box and leucine-rich repeat protein 5
GGDEF: diguanylate cyclase
HAMP: histidine kinases, adenyl cyclases, methyl-accepting proteins and phosphatases
MCP signal: methyl accepting chemotaxis protein
McHr: Methylococcus capsulatus hemerythrin
ML: maximum-likelihood
O_2_-binding hemerythrin: oxygen-binding hemerythrin
PDB: Protein Data Bank
pHMM: profile Hidden Markov Model
