# Supplementary figures and images for: Molecular Evolution of the Oxygen-Binding Hemerythrin Domain
Source: PLoS One. 2016 Jun 23;11(6):e0157904. doi: 10.1371/journal.pone.0157904 (PMC4919030; doi:10.1371/journal.pone.0157904)

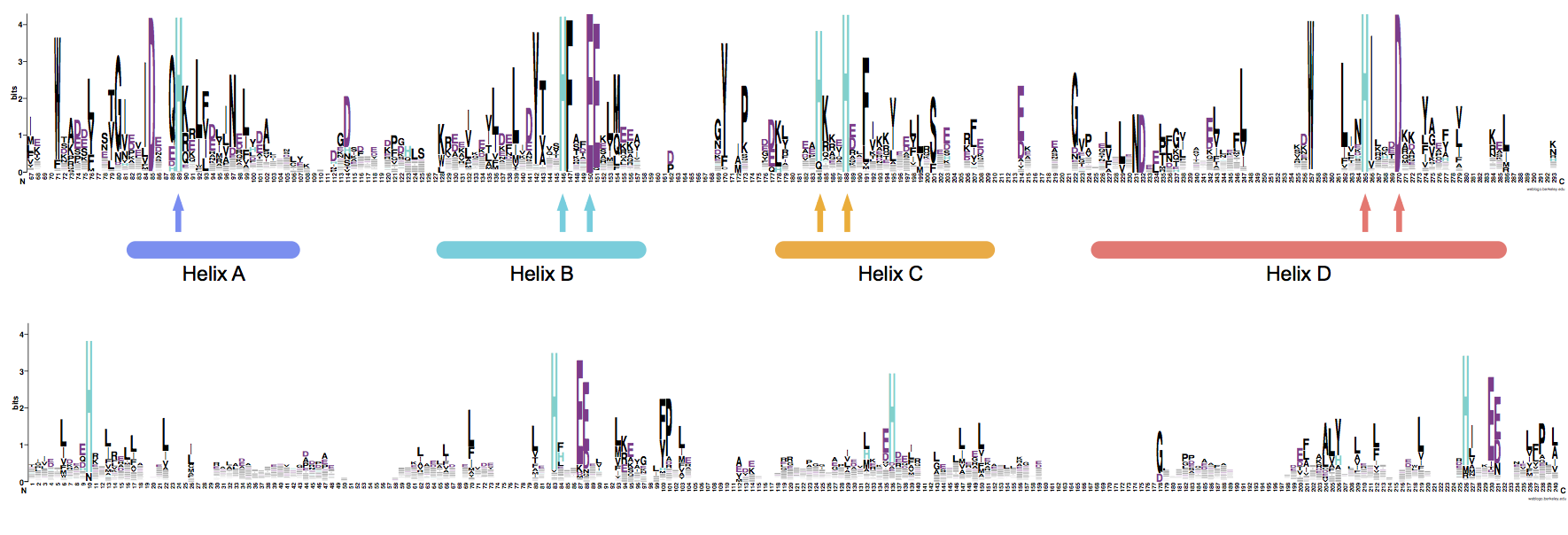

Supplement: S1 Fig — Logo representation of the multiple sequence alignment used as seed to calculate the profile Hidden Markov models. The vertical axis indicates the information content of a sequence position. The one-letter notation for amino acid sequences was used. Glutamic and aspartic acid (purple), histidine (cyan). (A) O2-binding Hr model. Positions of the iron-coordinating amino acids are indicated by arrows. Position and length of helical structures were predicted by Ali2D. (B) Pfam-A hemerythrin model. (TIF) [file pone.0157904.s001.tif]

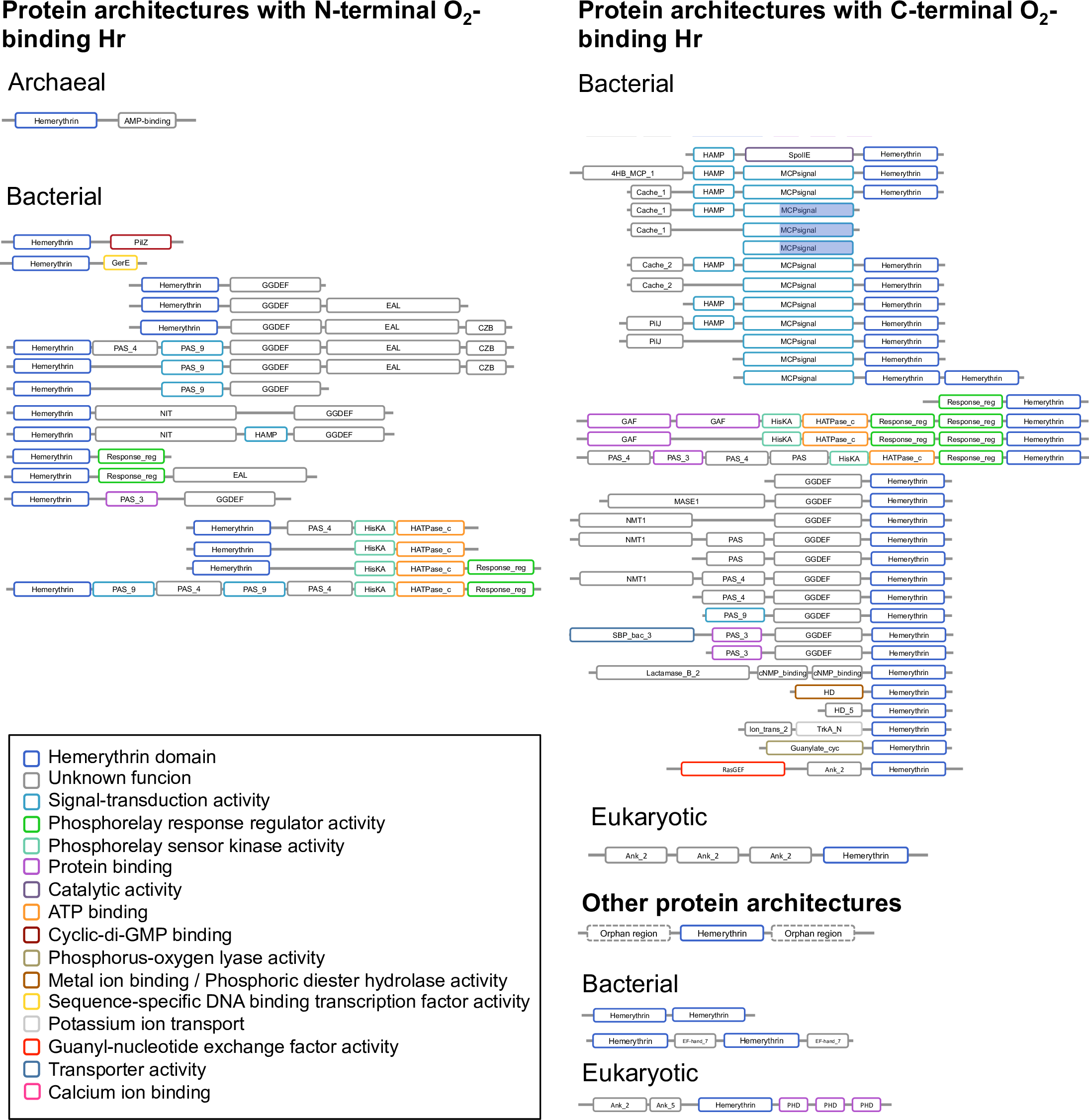

Supplement: S2 Fig — Protein domains identified in long O2-binding hemerythrin sequences are designated by their short Pfam-A family name. The number of sequences showing a particular architecture is indicated after a tabular space. (TIF) [file pone.0157904.s002.tif]

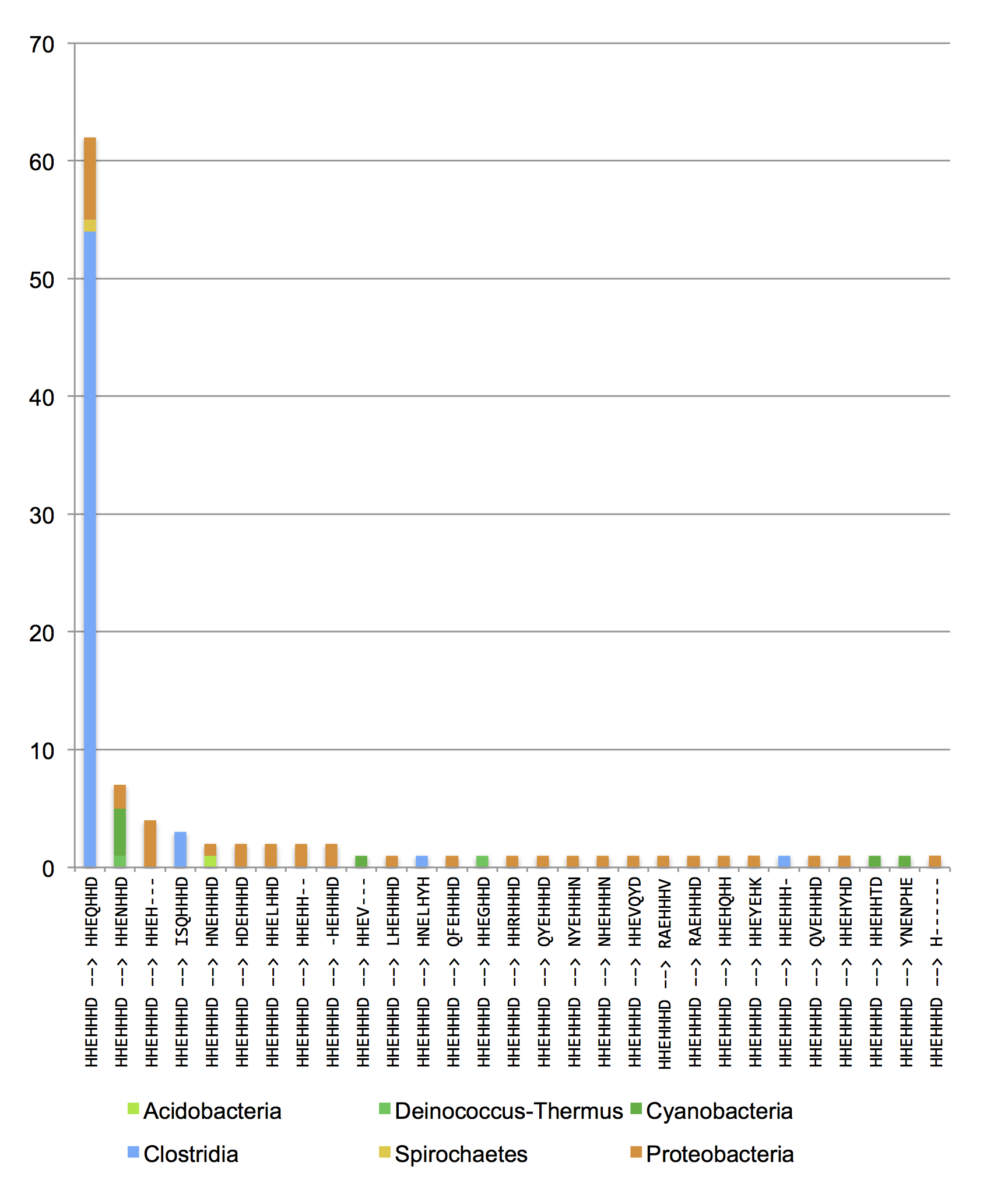

Supplement: S3 Fig — Phylogenetic tree based on a small subunit rRNA guide tree containing only completely sequenced species. Bacterial and archaeal species are collapsed on the phylum level. Eukaryotic species are collapsed together. n: number of species within the collapsed branch. The red bar is proportional to the number of species with at least one hemerythrin HHE cation-binding domain sequence in each group. The total number of genomes with at least one HHE cation-binding domain sequence is indicated by a purple number next to the bar. (TIF) [file pone.0157904.s003.tif]

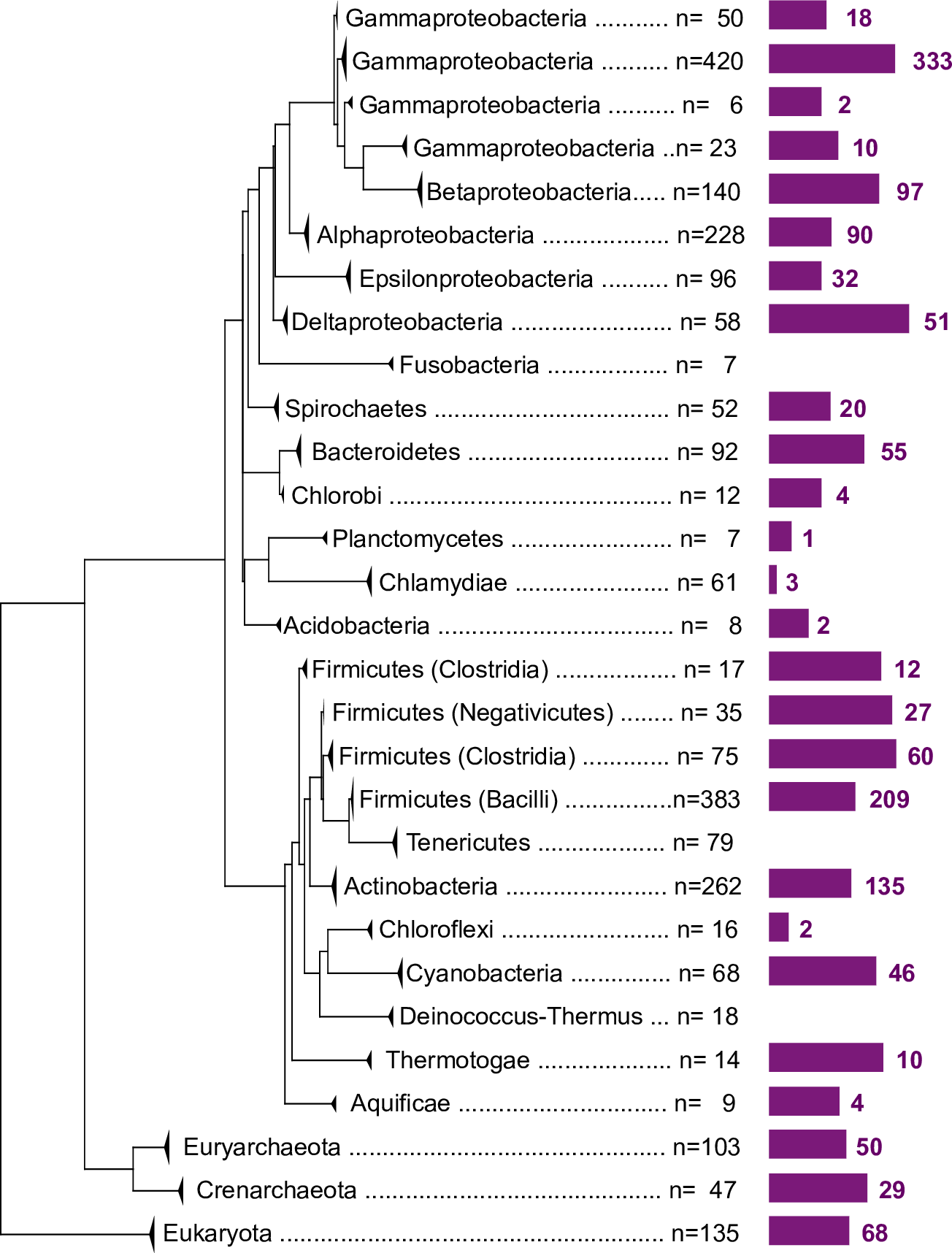

Supplement: S4 Fig — (TIF) [file pone.0157904.s004.tif]

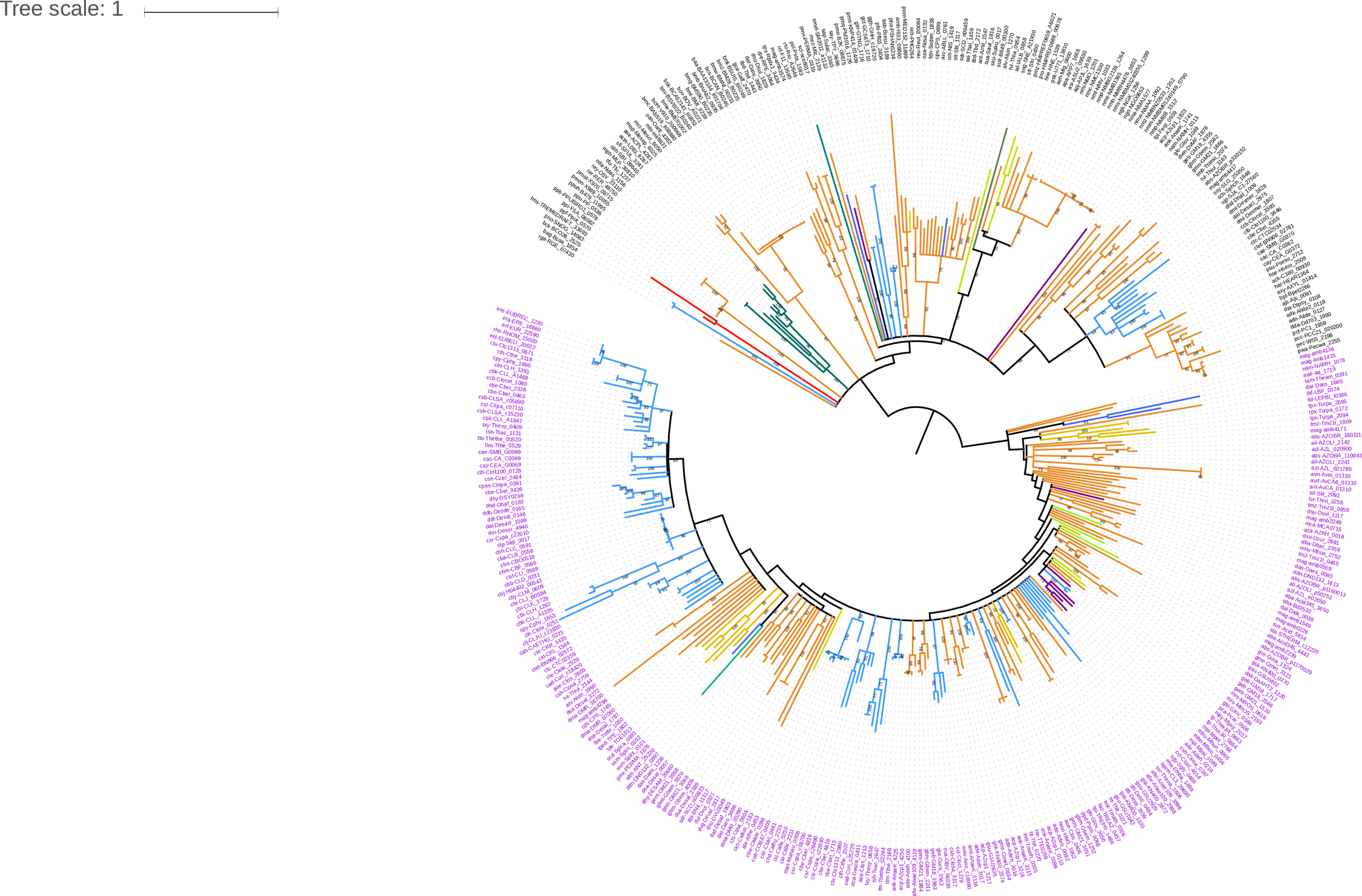

Supplement: S5 Fig — Sequences names are the same as in Fig 1. (TIF) [file pone.0157904.s005.tif]
